# Trend analysis of leprosy indicators in a hyperendemic Brazilian state, 2001–2015

**DOI:** 10.11606/S1518-8787.2019053000752

**Published:** 2019-07-24

**Authors:** Jefferson de Jesus Silva Anchieta, Léa Márcia Melo da Costa, Leonardo Costa Campos, Maurício dos Remédios Vieira, Osvaldina Silva Mota, Otaliba Libânio Morais, Marta Rovery de Souza, Rafael Alves Guimarães

**Affiliations:** I Secretaria do Estado da Saúde do Maranhão. São Luís, MA, Brasil; II Universidade Federal de Goiás. Instituto de Patologia Tropical e Saúde Pública. Goiânia, GO, Brasil

**Keywords:** Leprosy, Epidemiology, Communicable Disease Control, trends, Neglected Diseases, prevention & control, Time Series Studies, Hanseníase, Epidemiologia, Controle de Doenças Transmissíveis, tendências, Doenças Negligenciadas, prevenção & controle, Estudos de Séries Temporais

## Abstract

**OBJECTIVE:**

To analyze the temporal trend of leprosy indicators in a hyperendemic state of Brazil, from 2001–2015.

**METHODS:**

This is a time-series study of leprosy indicators in the state of Maranhão, Northeastern region of Brazil. The study used data from the Brazilian National System of Reportable Diseases, for the period between 2001 and 2015. The following indicators were evaluated: (i) detection coefficient in the general population; (ii) detection coefficient in people under 15 years old; (iii) rate of cases with grade 2 physical disability in the diagnosis; (iv) rate of examined contacts, and (v) proportion of healing . The Prais-Winsten regression model was used for trend analysis. Analyses were performed for the state and by each health region.

**RESULTS:**

77,697 leprosy cases were analyzed in the general population and 7,599 in individuals under 15 years old. The detection coefficient in the general population ranged from 80.7/100 thousand inhabitants in 2001 to 51.2/100 thousand inhabitants in 2015. The coefficient in the general population presented a downward trend (annual percentage variation [APV] = -2.98; 95%CI -4.15– -1.79). For the population under 15 years old, the rate was 24.9/100 thousand inhabitants in 2001, and 19.9/100 thousand inhabitants in 2015, with downward trend (APV = -3.07; 95%CI -4.95– -1.15). It was observed upward trend in rate of contacts examined (APV = 2.35; 95%CI 0.58–4.15) and rate of cases with grade 2 disability (APV = 2.19; 95%CI 0.23–4.19). Stationary trend was observed in the proportion of healing (APV = -0.10; 95%CI -0.50–0.30). Regional differences were found in the performance of the indicators.

**CONCLUSIONS:**

A downward trend for the detection coefficients in the general population and in individuals under 15 years old was found in Maranhão. Despite this result, the rates are still very high, demanding efforts from all spheres of public administration and health professionals to reduce the burden of the disease in the state.

## RESUMO

**OBJETIVO:**

Analisar a tendência temporal dos indicadores da hanseníase em estado brasileiro hiperendêmico, no período de 2011 a 2015.

**MÉTODOS:**

Trata-se de um estudo de série temporal dos indicadores de hanseníase no estado do Maranhão, região Nordeste, que utilizou dados do Sistema Nacional de Agravos de Notificação, no período de 2001 a 2015. Foram avaliados os seguintes indicadores: (i) coeficiente de detecção na população em geral; (ii) coeficiente de detecção em menores de 15 anos; (iii) proporção de casos com grau 2 de incapacidade física no diagnóstico; (iv) proporção de contatos examinados e (v) proporção de cura. Para análise da tendência foi utilizado o modelo de regressão de *Prais Winsten* . As análises foram realizadas para o Estado e por região de saúde.

**RESULTADOS:**

Foram analisados 77.697 casos de hanseníase na população geral e 7.599 em menores de 15 anos. O coeficiente de detecção na população geral variou de 80,7/100 mil habitantes em 2001 para 51,2/100 mil habitantes em 2015. Verificou-se tendência decrescente do coeficiente na população geral (variação percentual anual [VPA] = -2,98; IC95% -4,15– -1,79). Na população menor de 15 anos, o coeficiente foi de 24,9/100 mil habitantes em 2001 para 19,9/100 mil habitantes em 2015, com tendência decrescente (VPA = -3,07; IC95% -4,95– -1,15). Observou-se tendência crescente na proporção de contatos examinados (VPA = 2,35; IC95%: 0,58–4,15) e na proporção de casos com grau 2 de incapacidade (VPA = 2,19; IC95% 0,23–4,19). Verificou-se tendência estacionária na proporção de cura de hanseníase (VPA = -0,10; IC95% -0,50–0,30). Observaram-se diferenças regionais no desempenho dos indicadores.

**CONCLUSÕES:**

A tendência dos coeficientes de detecção na população geral e em menores de 15 anos mostrou-se decrescente no Maranhão. Apesar disso, as taxas ainda são muito elevadas, o que exige esforços de todas as esferas de gestão e profissionais da saúde para redução da carga da doença no estado.

## INTRODUCTION

Leprosy is a chronic infectious disease caused by the *Mycobacterium leprae* bacillus^1^ . This disease is characterized by dermatological and neurological effects and, although curable and despite the efforts made by government agencies in recent years through public policies, leprosy still represents a major problem for public health worldwide and in Brazil^2 , 3^ . This bacterium presents high infectivity and low pathogenicity, since it can infect large numbers of individuals, but very few grow sick^2^ .

Leprosy transmission occurs via the prolonged and intimate contact between susceptible or genetically predisposed individuals; untreated multibacillary patients; inhalation of microorganisms eliminated by superior airways; and to a lesser extent, direct contact. The nasal mucosa is the bacillus’ main entrance way^2 , 4^ . The best form of prevention and control of leprosy is through the detection and diagnosis of the infection, screening of family members, and early treatment^5^ .

*Mycobacterium leprae* infection still present high levels in many regions. In 2015, 210,758 leprosy cases were reported worldwide, and 136 countries reported cases of this infection^3^ . Of the total cases, 60.0% occurred in India (127,326), 13.0% in Brazil (26,395), and 8.0% in Indonesia (17,202). Thus, these countries comprised 81.0% of the new cases reported worldwide^3^ . The American continent has the second largest rate of cases globally. In 2015, 28,806 cases were reported in the Americas (13.0% of total cases). Of these cases, almost all were reported in Brazil (26,395; 91.6%), making it the country with the highest concentration of leprosy cases in the Americas^3^ .

In Brazil, leprosy still presents high morbidity and magnitude, causing disabilities and deformities which lead to further impairments in the clinical outcome due to the social stigma, loss of productivity and high costs for health services^3 , 6^ . In 2015, Brazil presented a general detection coefficient, a detection coefficient for people under 15 years old, and the detection coefficient with grade 2 disability of 14.06 cases, 4.28 cases and 0.91 cases/100 thousand inhabitants, respectively. Moreover, a 78.23% ratio of examined contacts, and a proportion of healing of 83.44%. Macro-region analyses found the highest detection coefficient for the general population in the Northern region (29.59/100 thousand inhabitants), and the lowest in the Southern region (3.49/100 thousand inhabitants) in 2015^7^ .

Three states have the highest detection coefficients in the general population of Brazil: Mato Grosso, Tocantins and Maranhão^7^ . In particular, 3,540 new cases were detected in Maranhão in 2015, corresponding to 13.40% of the cases in Brazil^8^ . In absolute numbers, Maranhão recorded more cases than eight of the 14 countries of the world with the highest rates of the disease. The detection coefficient in Maranhão was 51.27/100 thousand inhabitants in 2015^8^ . In the population under 15 years old, 375 cases were reported in 2015, representing a 17.5/100 thousand inhabitants detection rate. According to the standards set by the Brazilian Ministry of Health, Maranhão is considered hyperendemic place for leprosy in both indicators^8^ . The analyses of the indicators show that this state must be prioritized in the reduction of the burden of leprosy in Brazil.

Epidemiological indicators related to leprosy – such as the detection coefficient in the general population and in people under 15 years old, the ratio of new cases with grade 2 disability in the diagnosis, the ratio of contacts examined and the proportion of healing – allow the reach of the goal set by the World Health Organization (WHO) to be monitored, which is to reduce the burden of leprosy globally and locally^9^ . The systematic analysis of these indicators enables the evaluation of geographical and temporal variations of leprosy, contributing to the prevention and subsidizing the planning, administration and analysis of leprosy control interventions and policies, especially in Brazilian states with high leprosy rates, like Maranhão. In addition, there is a deficit of studies that evaluated the behavior, magnitude and trend of leprosy in Maranhão, which can subsidize the intensification of control actions of this infection^10^ . Given this context, this study sought to analyze the temporal trend of leprosy indicators in a hyperendemic state of Brazil, between 2001–2015.

## METHODS

### Study Design and Area

This is a time-series ecological study^11^ , using data from the Brazilian National System of Reportable Diseases ( *Sistema de Informação de Agravos de Notificação* – SINAN) of the state of Maranhão, from 2001 to 2015. Maranhão is located in the extreme northwest of the Northeastern region of Brazil, with a population of around 6,954,036 inhabitants (Brazilian Institute of Geography and Statistics, 2016). The state presents a 21 inhabitants/km^2^ demographic density and a R$7,852.71 per capita gross domestic product in 2011. The state’s area is 331,937,450 km^2^ , being divided into 217 municipalities, which are organized into 19 health regions. Maranhão is the second largest state in the Northeastern region and the eighth largest state of Brazil. Its capital São Luís is the most populous city in the state. About 24.3% of the population living in extreme poverty situation, thus occupying the 26^th^ position in the ranking of the Human Development Index (HDI) among Brazilian states, being the second worst. Furthermore, 64.5% of the municipalities of Maranhão present low HDI, where part of the population lives in vulnerability conditions.

Regarding the characterization of access and care from health services, by the end of 2015, the coverage of primary health care in the state was 85.9%, and 83.7% in the Family Health Strategy. In Maranhão, 1,006 basic health units (BHU) were enabled for fully providing leprosy care in 2015, i.e., those health units were capable of diagnosing and treating leprosy and conducting intra-household monitoring. This number corresponds to 62.5% of the total number of BHU registered in the state of Maranhão. Regarding reporting units by SINAN, there were 1,012 units, corresponding to 62.8% of the state total in the same period. Maranhão has two specialized units for the treatment of leprosy classified as grade 2, and one for grade 3, which perform preventive and corrective surgeries. These units are in the state capital.

### Data Source and Variables

This study analyzed the time-series of leprosy indicators of Maranhão, stratified by the 19 health regions in the period from 2001 to 2015, aiming to viewing the trend of leprosy and the monitoring of the state’s epidemiological and operational indicators. The data for the estimation of indicators were obtained from the SINAN database and population data from the Brazilian Institute of Geography and Statistics ( *Instituto Brasileiro de Geografia e Estatistica* – IBGE). Data from SINAN originate from compulsory notifications records, which consist of standardized forms with socio-demographic and clinical information, filled by health professionals^10^ .

The interest variables analyzed in this study were the indicators that represent the burden of morbidity and the magnitude of leprosy (leprosy detection rate in the general population and in people under 15 years old) and the quality of care services and prevention actions provided to patients (rate of intra-household contacts of new leprosy cases examined, the rate of cases with grade 2 in diagnosis and cure rate)^12^ .

Following the recommendations of the Ministry of Saúde^12^ , the following indicators were calculated for Maranhão:

(i) Detection coefficient of new leprosy cases (per 100,000 inhabitants):

Numerator: Number of new leprosy cases in residents.

Denominator: Total resident population in the established period.

Multiplication factor: 100 thousand.

The evaluation of this indicator enables the analysis of geographical and temporal variations in the distribution of new cases diagnosed, contributes to the prevention of infection and subsidizes the administration and analysis of public policies of leprosy control.

(ii) Detection coefficient in individuals under 15 years of age (per 100 thousand inhabitants):

Numerator: Number of new cases of leprosy in individuals under 15 years old.

Denominator: Resident population from zero to 14 years in the established period.

Multiplication factor: 100 thousand.

The detection coefficient among those under 15 years measures the strength of recent transmission of the endemic disease and its tendency.

(iii) Rate of leprosy cases with grade 2 disability at the time of diagnosis:

Numerator: Number of new confirmed cases of leprosy with grade 2 physical disability in residents.

Denominator: New cases with a degree of disability assessed, residents in the same place and time.

Multiplication factor: 100.

This indicator allows the evaluation of the effectiveness of the actions on the timely or early detection of leprosy cases.

(iv) Ratio of contacts examined:

Numerator: Number of contacts examined regarding new cases among residents during the cohort years.

Denominator: Total number of registered contacts related to new leprosy cases.

Multiplication factor: 100.

This indicator examines the capacity of health services in monitoring intra-household contacts of new leprosy cases, allowing the timely detection and the increase in the detection rate of infections.

(v) Proportion of healing in the cohort period

Numerator: New paucibacillary and multibacillary cases cured in the cohort years.

Denominator: New paucibacillary and multibacillary cases in the cohort years.

Multiplication factor: 100.

This indicator allows viewing the measures adopted to conduct the treatment in the planned period, measuring the quality of care offered to patients with leprosy.

### Statistical Analysis

Data were analyzed with the statistical program SPSS version 14.0. To describe the studied population, descriptive analysis was performed for all variables. Thus, the reported cases of leprosy were described by absolute and relative frequency by sex, age group, clinical form, operational classification and grade of disability.

The Prais-Winsten linear regression model was used for the time trend analysis^13^ . Initially, the logarithmic transformation of natural indicators was performed, which can reduce the heterogeneity of variance of the residuals from regression analysis. Following, the regression analysis was performed, considering the dependent variables “Y”, the analyzed indicators and the independent variable “x”, corresponding to the year of the study.

Thus, the linear regression equation can be described as:

Log(Y_t_) = β_0_ + β_1_x, where:

β_0_ is the constant or intercept;

Log(Yt), matches the value;

β_1_ is the linear trend coefficient;

*x* is the residual term.

After calculating the β coefficient and standard error (SE) in the regression analysis, the annual percentage variation and its 95% confidence interval (95%CI) were calculated using the following formulae:

$$\mathrm{APV}\;=\;-\;1\;+\;10^{\beta }\;\;$$

$$95\%\mathrm{CI}:\;-\;1+\;10^{\left(\beta \;+\;t*\mathrm{SE}\right)}$$

Trends were thus considered as upward, downward or stationary. The results were considered statistically significant when p < 0.05.

## RESULTS

### Descriptive Analysis of Cases

In the period from 2001 to 2015, 77,679 new leprosy cases were recorded in the state of Maranhão. Regarding the characteristics of the cases, most patients were male (57.7%) and had multibacillary operational classification (63.7%). Regarding the age groups, 9.8%, 35.8%, 42.1% and 12.3% of the cases occurred in individuals aged < 15 years, 15–34 years, 35–64 years and ≥ 65 years, respectively. Of the total cases, 19.2% and 6.3% presented grade 1 and 2 disability, respectively, at the time of diagnosis. The rate of cases in which disability grade was not assessed was 16.8%.

### Time Trends

#### General detection rate

The detection rate of leprosy in the general population of the state of Maranhão decreased from 88.9 cases/100 thousand inhabitants in 2001 to 64.61 cases/100 thousand inhabitants in 2015, showing a 27.32% (95%CI -27.49– -27.16%) decrease. The highest rate recorded during the period was observed in 2005 (103.57/100 thousand inhabitants). The Figure shows the ranking of the magnitude of the detection rate in the general population by health region between 2001 and 2015. There were differences in the distribution profile of rates among the regions. The Açailândia region presented a striking decrease, from the first position in 2001 to 13^th^ in 2015. The five regions with the largest percentage reduction in general detection rate were: Açailândia (-71.76%), Timon (-51.37%), Barra do Corda (-46.97%), Zé Doca (-46.84%) and Itapecuru Mirim (-44.87%). The Codó region presented a critical increase, moving from the 13^th^ position in 2001 for the second in 2015, an 50.91% increase. São Luís went from the 11^th^ position in 2001 for the sixth in 2015, a 7.51% increase.

**Figure:**
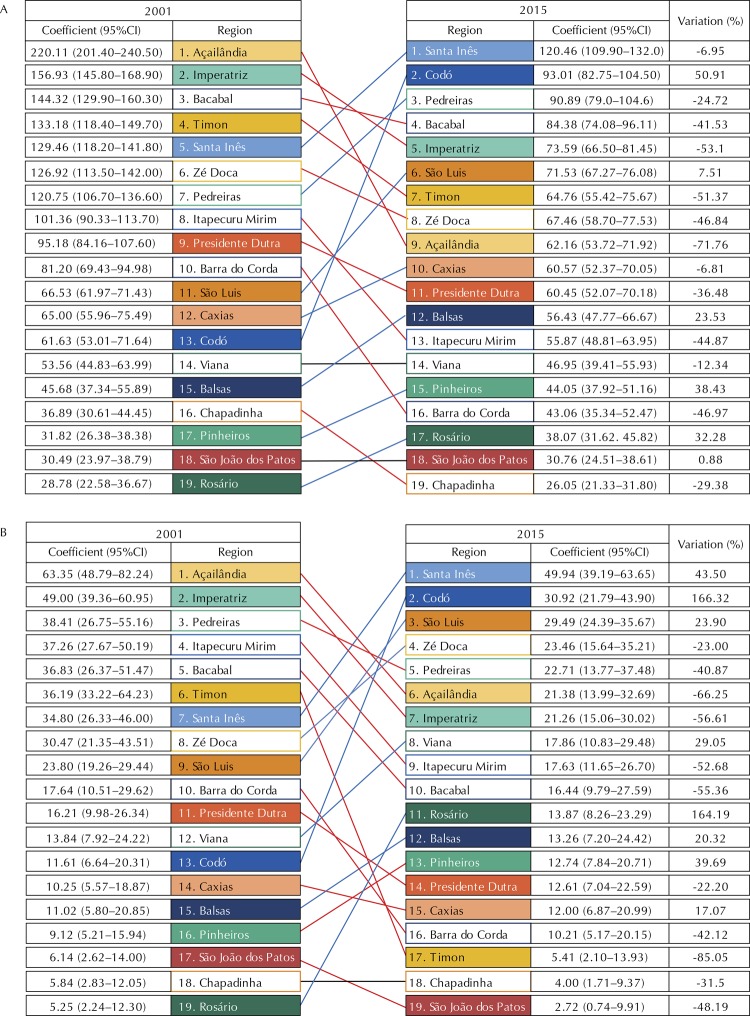
Ranking of general detection rate (A) and in individuals under 15 years (B) by health region of Maranhão between 2001 and 2015. Red lines indicate increase in the ranking; black lines, maintenance; and blue lines, decrease.

Table 1 shows the trend analysis of the analyzed indicators by health region. Maranhão presented a significant downward trend in the annual percentage variation (APV): -3.5% (95%CI -4.82– -2.15). Results stratified by health region also showed downward trend for the general detection rate in 11 (63.15%) health regions: Açailândia, Bacabal, Barra do Corda, Chapadinha, Imperatriz, Itapecuru Mirim, Pedreiras, Presidente Dutra, Santa Inês, Timon and Zé Doca. The other regions showed stability trend of this indicator (p > 0.05) ( Table 1 ).

**Table 1:** Trend of the general detection rate of leprosy (per 100 thousand inhabitants) by health region of Maranhão, Northeastern Brazil, 2001–2015.

Region	General detection rate				

	β (95%CI)	R^2^	p	Mean annual variation (95%CI)	Trend
Açailândia	-0.036 (-0.042– -0.030)	0.964	< 0.001	-9.46 (-10.85– -8.05)	↓
Bacabal	-0.023 (-0.031– -0.014)	0.847	< 0.001	-6.12 (-8.24– -3.96)	↓
Balsas	0.003 (-0.002–0.008)	0.214	0.234	0.89 (-0.63–2.44)	−
Barra do Corda	-0.019 (-0.032– -0.007)	0.519	0.005	-5.18 (-8.35– -1.89)	↓
Caxias	0.0005 (-0.005–0.006)	0.851	0.831	0.14 (-1.20–1.50)	−
Chapadinha	-0.011 (-0.016– -0.008)	0.822	< 0.001	-3.16 (-4.23– -2.08)	↓
Codó	0.008 (-0.003–0.0205)	-	0.126	2.42 (-0.73–5.67)	−
Imperatriz	-0.030 (-0.037– -0.023)	0.936	< 0.001	-7.92 (-9.54– -6.27)	↓
Itapecuru Mirim	-0.024 (-0.037– -0.011)	0.777	0.001	-6.33 (-9.64– -3.13)	↓
Pedreiras	-0.011 (-0.016– -0.006)	0.742	< 0.001	-3.15 (-4.48– -1.81)	↓
Pinheiros	-0.002 (-0.015–0.014)	0.269	0.681	-0.68 (-4.08–2.83)	−
Presidente Dutra	-0.007 (-0.013– -0.0004)	0.535	0.039	-1.90 (-3.62– -0.14)	↓
Rosário	0.007 (-0.004–0.018)	0.233	0.185	1.95 (-1.00–4.99)	−
Santa Inês	-0.013 (-0.025– -0.0009)	0.769	0.037	-3.51 (-6.31– -0.64)	↓
São João dos Patos	-0.002 (-0.013–0.008)	0.070	0.580	-0.78 (-3.66–2.19)	−
São Luís	-0.002 (-0.007–0.007)	0.953	0.953	-0.06 (-2.02–1.95)	−
Timon	-0.022 (-0.032– -0.011)	0.508	0.001	-5.80 (-8.38– -3.15)	↓
Viana	-0.008 (-0.023–0.006)	0.664	0.247	2.27 (-6.13–1.75)	−
Zé Doca	-0.020 (-0.026– -0.014)	0.964	< 0.001	-5.41 (-6.95– -3.84)	↓
Maranhão	-0.013 (-0.018– -0.007)	0.977	< 0.001	-3.49 (-4.82– -2.15)	↓

β: regression coefficient; R^2^: coefficient of determination; (↑): upward; (↓): downward; (–): stable

#### Detection rate in individuals under 15 years old

Between 2001 and 2015, 7,599 leprosy cases were recorded in individuals under 15 years old in the state of Maranhão. The coefficient decreased from 24.9 cases/100 thousand inhabitants in 2001 to 19.9 cases/100 thousand inhabitants in 2015, showing a 20.1% (95%CI -20.96– -19.20) decrease. The Figure shows the ranking of the detection rate in individuals under 15 years old by health region between 2001 and 2015. Açailândia dropped from the first place in 2001 to sixth place in 2015. The five regions with the largest percentage reduction in the detection rate in individuals under 15 years old were: Timon (-85.15%), Açailândia (-66.25%), Imperatriz (-56.61%), Bacabal (-52.68%) and Itapecuru Mirim (-52.68%). Again, Codó went from the 13^th^ position in 2001 to the second in 2015, a 116.3% increase. The Santa Inés region presented a striking rise, going from the seventh to the first position, a 43.50% increase. Moreover, Rosário presented a 164.19% increase in the detection rate. São Luís went from the ninth position in 2001 for the third in 2015, a 13.90% increase.

The state of Maranhão presented a significant downward trend in detection rate in individuals under 15 years old (APV = -3.60%; 95%CI -5.72– -1.43). Considering all regions, eight (42.1%) showed downward trend for this indicator: Açailândia, Bacabal, Barra do Corda, Chapadinha, Imperatriz, Itapecuru Mirim, Pedreiras, São João dos Patos and Timon; no region presented upward trend for this indicator; the other regions presented stability trend (p > 0.05) ( Table 2 ).

**Table 2:** Trend in the detection rate of leprosy in individuals under 15 years old (per 100 thousand inhabitants) by health region of Maranhão, Northeastern Brazil, 2001–2015.

Region	Detection rate in individuals < 15 years old				

	β (95%CI)	R^2^	p	Mean annual variation (95%CI)	Trend
Açailândia	-0.026 (-0.050– -0.002)	0.607	0.036	-6.85 (-12.70– -0.62)	↓
Bacabal	-0.028 (-0.036– -0.020)	0.843	< 0.001	-7.46 (-9.50– -5.37)	↓
Balsas	-0.007 (-0.015–0.001)	0.827	0.088	-1.94 (-4.14–0.30)	−
Barra do Corda	-0.029 (-0.056– -0.004)	0.266	0.029	-7.79 (-14.04– -1.08)	↓
Caxias	-0.002 (-0.014–0.009)	0.775	0.683	-0.62 (-3.71–2.57)	−
Chapadinha	-0.030 (-0.052– -0.009)	0.408	0.010	-7.92 (-13.12– -2.40)	↓
Codó	0.012 (-0.006–0.030)	0.250	0.180	3.34 (-1.64–8.58)	−
Imperatriz	-0.040 (-0.055– -0.026)	0.796	< 0.001	-10.49 (-13.88– -6.97)	↓
Itapecuru Mirim	-0.031 (-0.047– -0.015)	0.526	0.001	-8.25 (-12.14– -4.19)	↓
Pedreiras	-0.021 (-0.035– -0.007)	0.588	0.006	-5.67 (-9.18– -2.03)	↓
Pinheiros	-0.010 (-0.025–0.004)	0.140	0.158	-2.75 (-6.53–1.19)	−
Presidente Dutra	-0.013 (-0.031–0.004)	0.134	0.124	-3.66 (-8.21–1.12)	−
Rosário	0.013 (-0.013–0.040)	-	0.305	3.70 (-3.56–11.50)	−
Santa Inês	-0.013 (-0.029–0.002)	0.375	0.086	-3.65 (-7.68–0.54)	−
São João dos Patos	-0.014 (-0.029– -0.0009)	0.215	0.038	-3.98 (-7.51– -0.31)	↓
São Luís	0.002 (-0.011–0.015)	0.689	0.746	0.55 (-2.92–4.14)	−
Timon	-0.032 (-0.58– -0.007)	0.527	0.016	-8.44 (-14.47– -1.98)	↓
Viana	-0.003 (-0.019–0.012)	0.084	0.638	-0.97 (-5.15–3.39)	−
Zé Doca	-0.014 (-0.035–0.007)	0.504	0.166	-3.78 (-9.01–1.76)	−
Maranhão	-0.013 (-0.021– -0.005)	0.894	0.004	-3.60 (-5.72– -1.43)	↓

β: regression coefficient; R^2^: coefficient of determination; (↑): upward; (↓): downward; (–): stable

#### Rate of grade 2 physical disability

The state of Maranhão presented a significant upward trend in the rate of grade 2 disability (APV = 2.19%; 95%CI 0.23–4.19). Only three regions presented an upward trend of stability for this indicator (15.78%): Balsas, Santa Inês and São Luís. Almost all regions (16/82, 20%) presented a trend of stability for this indicator (p > 0.05) ( Table 3 ).

**Table 3:** Trend of the indicator rate of cases with grade 2 disabilities by health region of Maranhão, Northeastern Brazil, 2001–2015.

Region	Rate of cases with grade 2 disability				

	β (95%CI)	R^2^	p	Mean annual variation (95%CI)	Trend
Açailândia	0.018 (-0.007–0.437)	0.174	0.139	5.12 (-1.75–12.47)	−
Bacabal	0.027 (0.012–0.041)	0.424	0.001	7.59 (3.54–11.80)	↑
Balsas	0.018 (-0.039–0.076)	0.235	0.506	5.08 (-9.95–22.61)	−
Barra do Corda	0.004 (-0.025–0.335)	0.077	0.774	1.09 (-6.59–9.41)	−
Caxias	-0.004 (-0.018–0.011)	0.143	0.597	-1.00 (-4.86–3.01)	−
Chapadinha	-0.009 (-0.009– -0.027)	0.251	0.290	2.55 (-2.32–7.67)	−
Codó	-0.014 (-0.036–0.007)	0.262	0.171	-3.87 (-9.30–1.88)	−
Imperatriz	0.008 (-0.002–0.018)	0.284	0.097	2.23 (-0.42–4.95)	−
Itapecuru Mirim	0.012 (-0.003–0.027)	0.372	0.108	3.39 (-0.78–7.74)	−
Pedreiras	-0.003 (-0.028–0.022)	0.328	0.814	-0.77 (-7.35–6.27)	−
Pinheiros	-0.019 (-0.042–0.002)	0.200	0.073	-5.26 (-10.69–0.50)	−
Presidente Dutra	-0.0002 (-0.013–0.012)	0.375	0.963	-0.08 (-3.46–3.43)	−
Rosário	-0.016 (-0.045–0.014)	0.074	0.282	-4.17 (-11.61–3.90)	−
Santa Inês	0.026 (0.005–0.047)	0.456	0.016	7.43 (1.65–13.53)	↑
São João dos Patos	-0.008 (-0.048–0.031)	0.269	0.654	-2.26 (-12.11–8.70)	−
São Luís	0.012 (0.005–0.018)	0.593	0.002	3.31 (1.52–5.13)	↑
Timon	0.002 (-0.063–0.066)	-	0.957	0.45 (-15.54–19.47)	−
Viana	0.008 (-0.024–0.403)	0.103	0.597	2.22 (-6.22–11.41)	−
Zé Doca	-0.010 (-0.028–0.007)	0.075	0.211	-2.84 (-7.27–1.80)	−
Maranhão	0.008 (0.0007–0.015)	0.721	0.033	2.19 (0.23–4.19)	↑

β: regression coefficient; R^2^: coefficient of determination; (↑): upward; (↓): downward; (–): stable

#### Rate of contacts examined

During the analysis period, Maranhão showed an upward trend in the rate of contacts examined (APV = 2.8%; 95%CI 0.75–4.83). Among all regions, 14 (73.68%) presented stationary trend (p > 0.05). The study found an upward trend in the rate of contacts examined in only four (21.05%) health regions: Açailândia, Codó, Imperatriz and Pedreiras. Balsas presented a downward trend for this indicator ( Table 4 ).

**Table 4:** Trend of the rate of contacts examined by health region of Maranhão, Northeastern Brazil, 2001–2015.

Region	Ratio of contacts examined				

	β (95%CI)	R^2^	p	Mean annual variation (95%CI)	Trend
Açailândia	0.028 (0.013–0.042)	0.768	0.001	7.89 (3.73–12.22)	↑
Bacabal	0.016 (-0.035–0.068)	0.652	0.511	4.51 (-9.05–20.10)	−
Balsas	-0.012 (-0.023– -0.002)	0.916	0.026	-3.44 (-6.22– -0.57)	↓
Barra do Corda	-0.0034 (-0.016–0.009)	0.900	0.545	-0.94 (-4.10–2.32)	−
Caxias	-0.001 (-0.016–0.012)	0.936	0.780	-0.50 (-4.14–3.27)	−
Chapadinha	0.003 (-0.024–0.029)	0.789	0.882	0.77 (-6.14–8.18)	−
Codó	0.019 (0.095–0.021)	0.937	0.008	5.32 (1.68–9.08)	↑
Imperatriz	0.010 (0.0009–0.019)	0.901	0.034	2.82 (0.29–5.42)	↑
Itapecuru Mirim	0.014 (-0.007–0.037)	0.395	0.170	4.12 (-1.87–10.49)	−
Pedreiras	0.017 (0.002–0.032)	0.543	0.029	4.79 (0.64– 9.11)	↑
Pinheiros	0.0009 (-0.029–0.031)	0.491	0.947	0.26 (-7.63–8.83)	−
Presidente Dutra	0.003 (-0.015–0.020)	0.923	0.739	0.77 (-3.96–5.73)	−
Rosário	-0.002 (-0.023–0.019)	0.665	0.837	-0.57 (-6.18–5.38)	−
Santa Inês	0.029 (-0.006–0.065)	-	0.101	8.24 (-1.63–19.11)	−
São João dos Patos	0.003 (-0.020–0.279)	0.438	0.748	1.00 (-5.33–7.76)	−
São Luís	0.002 (-0.012–0.017)	-	0.710	0.72 (-3.25–4.86)	−
Timon	0.020 (-0.019–0.060)	0.718	0.289	5.66 (-4.94–17.43)	−
Viana	0.010 (-0.005–0.026)	0.879	0.178	2.87 (-1.38–7.31)	−
Zé Doca	-0.002 (-0.027–0.023)	0.871	0.847	-0.63 (-7.19–6.40)	−
Maranhão	0.010 (0.002–0.017)	0.969	0.012	2.77 (0.75–4.83)	↑

β: regression coefficient; R^2^: coefficient of determination; (↑): upward; (↓): downward; (–): stable

#### Proportion of healing

Maranhão presented a stationary trend in the proportion of healing of leprosy during the analyzed period (VPA = -0.12%; 95%CI -0.58–0.34). Fourteen (73.7%) health regions presented stability trend (p < 0.05) in the proportion of healing between 2001 and 2015. Balsas, Imperatriz and Pedreiras presented upward trend, and Itapecuru Mirim and Pinheiros presented downward trend ( Table 5 ).

**Table 5:** Trend of the indicator proportion of healing by health region of Maranhão, Northeastern Brazil, 2001–2015.

Region	Cure rate				

	β (95%CI)	R^2^	p	Mean annual variation (95%CI)	Trend
Açailândia	-0.0003 (-0.004–0.005)	0.996	0.894	0.63 (0.31–0.94)	−
Bacabal	0.001 (-0.004–0.006)	0.988	0.641	0.33 (-1.15–1.83)	−
Balsas	0.002 (0.001–0.003)	0.974	0.001	0.63 (0.31–0.94)	↑
Barra do Corda	0.002 (-0.002–0.006)	0.982	0.335	0.48 (-0.40–1.36)	−
Caxias	-0.0004 (-0.002–0.001)	0.979	0.548	-0.12 (-0.54–0.31)	−
Chapadinha	0.002 (-0.0009–0.005)	-	0.157	0.59 (-0.35–1.54)	−
Codó	-0.0007 (-0.004–0.002)	0.994	0.605	-0.19 (-0.77–0.39)	−
Imperatriz	0.001 (0.0002–0.003)	0.989	0.024	0.38 (0.10–0.66)	↑
Itapecuru Mirim	-0.002 (-0.003– -0.001)	0.984	< 0.001	-0.52 (-0.75– -0.29)	↓
Pedreiras	0.004 (0.002–0.006)	0.992	0.001	1.10 (0.41–1.80)	↑
Pinheiros	-0.006 (-0.008– -0.004)	0.998	< 0.001	-1.68 (-2.22– -1.14)	↓
Presidente Dutra	-0.0006 (-0.003–0.002)	0.906	0.682	-0.15 (-0.88–0.47)	−
Rosário	0.0006 (-0.005–0.006)	-	0.813	0.18 (-1.24–1.61)	−
Santa Inês	0.0001 (-0.003–0.003)	0.996	0.893	0.05 (-0.40–0.50)	−
São João dos Patos	-0.0009 (-0.005–0.003)	0.956	0.664	-0.26 (-1.38–0.87)	−
São Luís	-0.003 (-0.005– -0.0006)	0.987	0.018	-0.88 (-1.76–0.00)	−
Timon	0.001 (-0.0003–0.003)	0.996	0.111	0.35 (-0.03–0.73)	−
Viana	-0.002 (-0.007–0.002)	0.734	0.236	-0.68 (-1.84–0.49)	−
Zé Doca	-0.006 (-0.003–0.019)	0.997	0.621	-0.16 (-0.82–0.51)	−
Maranhão	-0.0004 (-0.002–0.001)	0.999	0.580	-0.12 (-0.58–0.34)	−

β: regression coefficient; R^2^: coefficient of determination; (↑): upward; (↓): downward; (–): stable

## DISCUSSION

This study sought to analyze time trends and the performance of leprosy indicators in the state of Maranhão, Northeastern Brazil. The results showed a trend of reduction of the coefficient of general detection and detection in individuals under 15 years old during the analyzed period, thus following the national trend. However, these two indicators presented diverse patterns among the different health regions. The rate of contacts examined increased in the state as a whole, but remained stable in most regions, which was a similar pattern to the indicator rate of cases with grade 2 disability at the time of diagnosis. The proportion of healing showed stability over the analyzed period in the whole state and in most health regions. Performance analysis of the indicators showed the hyperendemic character of the detection coefficient in the general population in the state and in most regions. Regarding the detection coefficient in individuals under 15 years old, a very high pattern was identified for the state and hyperendemic for most regions. In the state and most health regions, the rate of cases with grade 2 disability showed average performance; proportion of healing, regular; and rate of contacts examined, precarious.

Studies have shown downward trend for the detection rate of leprosy in the general population in Brazil and in other hyperendemic states in recent years^8 , 14^ . Despite the time difference with this study, when analyzing the detection coefficient of leprosy disease in the general population of Brazil between 1980 and 2006, Penna et al.^15^ identified a downward trend for this indicator from 2003 onwards (p < 0.001). In Fortaleza (Ceará, Northeastern region of Brazil), a study showed downward trend for the general detection rate, with -4.0% APV (95%CI -5.6– -2.3)^8^ . In the state of Tocantins (Northern region), Monteiro et al.^16^ found a downward trend for the general detection coefficient between 2001 and 2012 (p = 0.025). An ecological study in municipalities with high risk for leprosy transmission of the states of Mato Grosso (Midwest region), Tocantins, Rondônia, Pará (Northern region) and Maranhão (Northeastern region), observed reduction in the incidence rate from 89.10 to 56.98 per 100 thousand inhabitants between 2001 and 2012, with -4.2% APV (95%CI -5.9– -2.4)^14^ . The results of this study also showed a downward trend for the general detection coefficient, suggesting a decrease in strength of leprosy in Maranhão^8^ . However, the state still presents a hyperendemic pattern, a severe public health problem. Moreover, we found significant differences in the detection coefficient in the general population among health regions. Of the 19 regions of the Maranhão, most (n = 11) presented downward trend for the general coefficient, four stability trends and no downward trend of this indicator. The health regions that presented the highest downward trends for this coefficient were Açailândia and Imperatriz.

To consider the strength of morbidity, represented by the magnitude and trend of leprosy found in the population of children and adolescents, is crucial for the planning of infection control actions^17^ . The detection of new cases in individuals under 15 years suggests successive transmission, persistence or active circulation of *Mycobacterium leprae* , and the lack of effective control measures for leprosy^18 , 19^ . For this indicator, this study showed a stationary trend in most health regions and downward trend in eight of them, corroborating another study in Brazil^18^ . However, this indicator remains very high in Maranhão and hyperendemic in almost all regions, indicating the need for increasing control measures among this population.

The differences between the results of the indicators mentioned in health regions suggest the need for the assessment of economic and risk factors, and of the actions in each health region; thus, seeking to reduce the burden of leprosy in Maranhão. This will allow the discrepancies in the results found to be better explained and could contribute to the intensification of control and prevention actions that consider regional particularities.

The downward trend of the detection coefficient indicators in the general population and in individuals under 15 years possibly reflects the intensification of leprosy control actions in Maranhão in recent years. Among the actions, we can highlight the expansion of multidrug therapy (MDT) for diagnosed patients, early detection of new cases, *Bacillus Calmette-Guerin* (BCG) vaccination of patients’ contacts, training of health professionals by seeking to improve the decentralization of the leprosy control program, active search campaigns in schools, and monitoring and support to innovative actions in the municipalities with the highest infection burden levels. For example, between 2002 and 2016, 5,040 health professionals received training. Detection campaigns conducted in schools were held in 77 (35.4%) municipalities in 2013 and in 162 (74.6%) in 2016, a 47.5% growth. During this period, 203,329 students were examined, and 192 cases were detected in individuals under 15 years in Maranhão. The state has also been supporting the priority municipalities with innovative actions such as actively searching new cases of leprosy and searching intra- and extra-household contacts of cases diagnosed in the last three or five years. Moreover, monitoring visits were expanded to the municipalities and the campaigns and joint efforts were spread across the state. In 2016, the population underwent 10,203 consultations in 71 municipalities, with 271 cases diagnosed. In 2017, the numbers were 13,849 consultations, 1,032 suspected and 168 new cases detected in 64 municipalities.

The risk of a healthy individual contracting leprosy increases ninefold with prolonged contact at home^20 , 21^ . Therefore, conducting examinations of intra-household contacts of all new diagnosed cases is crucial for the prevention and control of leprosy, as it allows the early diagnosis and contributes to the prevention of subsequent deformities and disabilities^18 , 20 , 22^ . In Brazil, Decree 3,125 from October 27, 2010, of the Ministry of Health considers as intra-household contact any individual who lives or used to live with an individual diagnosed with leprosy in the past five years^23^ . In recent years, the Ministry of Health reworked the concept and began to consider household contact as any individual who lives or used to live with a leprosy patient, and social contact as any individual who has contact or used to have contact in a close and prolonged manner, regardless of being a family relationship or not^12^ . Social contacts such as neighbors, work or school colleagues, must be investigated according to the degree and type of contact (i.e., if they had very close and prolonged contact with the untreated patient). However, the evaluation focus must be on patient’s relatives contacts (parents, siblings, grandparents, uncles and aunts, among others), or other individuals with prolonged contact^12^ . Regarding the ratio of examined contacts indicator, most regions presented stationary trend, and only four showed an upward trend. Balsas presented a downward trend for this indicator ( Table 4 ). Stationary or upward trends of this indicator imply improvements in actions and activities of epidemiological monitoring^17^ . Therefore, these results show the need for further efforts in all health regions of Maranhão to increase the rate of examined contacts, consequently improving this indicator in the state.

The most effective way to prevent the physical disabilities arising from the leprosy is the early diagnosis combined with the treatment of the disease and its reactions^24 , 25^ . The WHO estimates that the diagnosis and early treatment of leprosy infections led to the prevention of physical disability in approximately four million people worldwide^24^ . In this study, we observed an upward trend in the rate of grade 2 disability at the time of diagnosis in Maranhão, and most regions presented stability trends; thus, corroborating other studies conducted in Brazil^8 , 14^ . Only three regions showed upward trend for this indicator. The stationary trend in the rate of grade 2 physical disability indicates the late diagnosis of cases and failures in preventing physical disabilities^8 , 14^ . Similarly, the upward trend in some health regions indicates late identification, contributing to the persistence of hidden prevalence (undiagnosed cases) and the consequent increase of transmissibility^26^ . On the other hand, a stationary trend or increase in this indicator may suggest improvements in the evaluation of disabilities by health professionals. Given this context, increasing the control and intensifying prevention strategies of grade 2 disability of leprosy are required for reducing the disease burden in the state^14^ . Mass campaigns for the diagnosis of leprosy must be intensified in all health regions of Maranhão, integrating them with other health programs for disease control, and establishing the decrease in the grade of disability at the time of the diagnosis as the objective^27^ .

The effectiveness of leprosy treatment is evaluated by the proportion of healing of the patients in the years of the cohort^12^ . It corresponds to an indicator for evaluating the quality of patient care and operation of the leprosy control program^23^ . Since 1985, the multidrug therapy (MDT) against leprosy has been made available free of charge worldwide, which significantly reduced the disease’s burden^24 , 28^ . Leprosy therapy, in Brazil, is conducted in an outpatient manner, using standardized therapeutic schemes according to the operational classification of the infection. The results found in this study show a stationary trend for the proportion of healing in Maranhão and most of its regions, in addition to an upward trend in three regions, suggesting the efficacy of MDT. However, two regions (Itapecuru Mirim and Pinheiros) showed downward trend for this indicator. This finding suggests the need for reorganization of the care services for leprosy patients in these regions, seeking to reduce the abandonment of therapy and ensure discharge for cure.

Our study presents some limitations. First, we used secondary data for the analysis, which suffer bias in the quality and quantity of information^16^ . Thus, underreporting of cases during the analyzed period may have occurred due to the lack of completion of notification forms or errors during while transferring the information^29^ . Nevertheless, this study showed the epidemiological scenario of the leprosy indicators in Maranhão. Moreover, we made advances when compared to previous studies, since we analyzed the trends and the performance of the indicators by health region of the state. These analyses allow a study focused on regionalities of Maranhão. Studies conducted in other states of Brazil did not analyze the trend by region, failing to obtain important data to subsidize specific control actions.

In conclusion, despite the reduction of detection coefficients in the general population and in individuals under 15 years old in Maranhão, these indicators remain high in many regions, which still assigns the state a hyperendemic character for this infection. The rate of grade 2 physical disability at the time of diagnosis is also high in almost all regions. The results of this study suggest the need for the intensification of efforts and actions to eliminate this disease, especially in regions with high magnitude for leprosy. Our results also suggest the expansion of leprosy control measures in the state, including the increase in the rate of contacts examined, the promotion of the early detection of leprosy cases through active search and detection campaigns of cases in schools and vulnerable regions, ensuring the onset and adherence to treatment to increase the proportion of healing and decrease of transmissibility rates, improvements in the prevention of physical disabilities, among others. Finally, subsequent studies must be conducted to verify the quality of leprosy control actions in the regions to better understand the regional differences of indicators.

## References

[1] White C, Franco-Paredes C (2015). Leprosy in the 21st century. Clin Microbiol Rev.

[2] Lastória JC, Abreu MAMM (2014). Leprosy: review of the epidemiological, clinical, and etiopathogenic aspects - Part 1. An Bras Dermatol.

[3] World Health Organization (2016). Global leprosy update, 2015 :time for action, accountability and inclusion. Wkly Epidemiol Rec.

[4] Bratschi MW, Steinmann P, Wickenden A, Gillis TP (2015). Current knowledge on Mycobacterium leprae transmission: a systematic literature review. Lepr Rev.

[5] Rodrigues LC, Lockwood DNJ (2011). Leprosy now: epidemiology, progress, challenges, and research gaps. Lancet Infect Dis.

[6] Silva CLM, Fonseca SC, Kawa H, Palmer DOQ (2017). Spatial distribution of leprosy in Brazil: a literature review. Rev Soc Bras Med Trop.

[7] Brito AL, Monteiro LD, Ramos AN, Heukelbach J, Alencar CH (2016). Temporal trends of leprosy in a Brazilian state capital in Northeast Brazil: epidemiology and analysis by joinpoints, 2001 to 2012. Rev Bras Epidemiol.

[8] Ministério da Saúde (BR) Sala de Apoio à Gestão Estratégica -SAGE.

[9] Organização Mundial da Saúde, Escritório Regional para o Sudeste Asiático (2016). Estratégia mundial de eliminação da lepra 2016-2020: acelerar a ação para um mundo sem lepra.

[10] Monteiro LD, Martins-Melo FR, Brito AL, Alencar CH, Heukelbach J (2015). Padrões espaciais da hanseníase em um estado hiperendêmico no Norte do Brasil, 2001-2012. Rev Saude Publica.

[11] Bhaskaran K, Gasparrini A, Hajat S, Smeeth L, Armstrong B (2013). Time series regression studies in environmental epidemiology. Int J Epidemiol.

[12] Ministério da Sáude (BR), Secreteria de Vigilância em Saúde, Departamento de Vigilância das Doenças Transmissíveis (2016). Diretrizes para vigilância, atenção e eliminação da hanseníase como problema de saúde pública: manual técnico-operacional.

[13] Antunes JLF, Cardoso MRA (2015). Uso da análise de séries temporais em estudos epidemiológicos. Epidemiol Serv Saude.

[14] Freitas LRS, Duarte EC, Garcia LP (2016). Trends of main indicators of leprosy in Brazilian municipalities with high risk of leprosy transmission, 2001-2012. BMC Infect Dis.

[15] Penna MLF, Oliveira MLW, Carmo EH, Penna GO, Temporão JG (2008). The influence of increased access to basic healthcare on the trends in Hansen’s disease detection rate in Brazil from 1980 to 2006. Rev Soc Bras Med Trop.

[16] Monteiro LD, Martins-Melo FR, Brito AL, Lima MS, Alencar CH, Heukelbach J (2015). Tendências da hanseníase no Tocantins, um estado hiperendêmico do Norte do Brasil, 2001-2012. Cad Saude Publica.

[17] Veira MCA, Nery JS, Paixão ES, Andrade KVF de, Penna GO, Teixeira MG (2018). Leprosy in children under 15 years of age in Brazil: A systematic review of the literature. PLoS Negl Trop Dis.

[18] Oliveira MBB, Diniz LM (2016). Leprosy among children under 15 years of age: literature review. An Bras Dermatol.

[19] Chaitra P, Bhat RM (2013). Postelimination status of childhood leprosy: report from a tertiary-care hospital in South India. Biomed Res Int.

[20] Beers SM, Hatta M, Klatser PR (1999). Patient contact is the major determinant in incident leprosy: implications for future control. Int J Lepr Other Mycobact Dis.

[21] Moet FJ, Pahan D, Schuring RP, Oskam L, Richardus JH (2006). Physical distance, genetic relationship, age, and leprosy classification are independent risk factors for leprosy in contacts of patients with leprosy. J Infect Dis.

[22] Sales AM, Ponce de Leon A, Düppre NC, Hacker MA, Nery JAC, Sarno EN (2011). Leprosy among patient contacts: a multilevel study of risk factors. PLoS Negl Trop Dis.

[23] Ministério da Saúde (BR) (2010). Portaria Nº 3.125, de 7 de outubro de 2010. Aprova as Diretrizes para Vigilância, Atenção e Controle da Hanseníase.

[24] World Health Organization (2011). World Report On Disability.

[25] Santos VS, Matos AMS, Oliveira LSA, Lemos LMD, Gurgel RQ, Reis FP (2015). Clinical variables associated with disability in leprosy cases in northeast Brazil. J Infect Dev Ctries.

[26] Oliveira KS, Souza J, Campos RB, Zilly A, Silva-Sobrinho RA (2015). Avaliação dos indicadores epidemiológicos e operacionais para a hanseníase em municípios prioritários no estado do Paraná, 2001 a 2010. Epidemiol Serv Saude.

[27] Monteiro LD, Martins-Melo FR, Brito AL, Alencar CH, Heukelbach J (2015). Physical disabilities at diagnosis of leprosy in a hyperendemic area of Brazil: trends and associated factors. Lepr Rev.

[28] Suzuki K, Akama T, Kawashima A, Yoshihara A, Yotsu RR, Ishii N (2012). Current status of leprosy: epidemiology, basic science and clinical perspectives. J Dermatol.

[29] Freitas BHBM, Cortela DCB, Ferreira SMB (2017). Tendência da hanseníase em menores de 15 anos em Mato Grosso (Brasil), 2001-2013. Rev Saude Publica.

